# Revue narrative des stratégies de prise en charge des morsures de serpent en Afrique de l’Ouest francophone

**DOI:** 10.48327/mtsi.v6i3.2026.834

**Published:** 2026-08-28

**Authors:** Absa LAM, Khardiata DIALLO, Moussa DIALLO, Arame KANE, Yacine NDIONE, Coumba Ndoffene DIOUF, Mamadou FALL

**Affiliations:** 1 Centre antipoison du Senegal, Centre hospitalier national universitaire (CHNU) de Fann, Dakar, Senegal; 2 Universite Cheikh Anta Diop de Dakar, Senegal; 3 Service des maladies infectieuses et tropicales, Centre hospitalier national universitaire (CHNU) de Fann, Dakar, Senegal

**Keywords:** Morsure de serpent, Envenimation, Antivenin, Prise en charge, Afrique de l’Ouest francophone, Afrique subsaharienne, Snakebite, Envenomation, Antivenom, Management, Francophone West Africa, Sub-Saharan Africa

## Abstract

**Introduction:**

En Afrique de l’Ouest, les envenimations par morsure de serpent constituent un problème de santé publique sousestimé, particulièrement en milieu rural où l’accès aux soins est limité et les pratiques traditionnelles prédominent. Face aux disparités dans l’accès aux soins et de prise en charge et au manque de protocoles standardisés, une revue narrative vise à synthétiser les stratégies de prise en charge des morsures de serpent en Afrique de l’Ouest selon les circonstances.

**Matériel et méthode:**

Une revue narrative qualitative a été réalisée à partir de publications scientifiques et rapports publiés entre 1997 et 2023. La sélection des études s’est appuyée sur des critères d’inclusion stricts portant sur les modalités thérapeutiques dans certains pays d’Afrique de l’Ouest francophone.

**Résultats:**

La prise en charge des envenimations est freinée par le recours prioritaire aux tradipraticiens, un accès limité aux soins modernes (antivenins coûteux, stocks irréguliers, protocoles non unifiés).

**Discussion/conclusion:**

La prise en charge des morsures de serpent en Afrique de l’Ouest reste hétérogène et globalement insuffisante. Les coûts indirects pour les victimes restent peu pris en compte. Il est indispensable de renforcer l’accessibilité aux antivenins adaptés, de former les professionnels de santé, de structurer les systèmes de surveillance et d’intégrer davantage les populations dans des campagnes de sensibilisation adaptées à chaque contexte local.

## Abstract (en)

**Introduction:**

In West Africa, snakebite envenomation is an underestimated public health problem, particularly in rural areas where access to care is limited and traditional practices are common. Due to disparities in access to care and treatment, as well as a lack of standardized protocols, this narrative review aims to synthesize strategies for managing snakebites in West Africa according to specific circumstances.

**Materials and methods:**

A qualitative narrative review was conducted using scientific publications and reports published between 1997 and 2023. Study selection was based on strict inclusion criteria focusing on treatment modalities in certain French-speaking West African countries.

**Results:**

The management of snakebite envenomation is hindered by reliance on traditional healers and limited access to modern care, including expensive antivenoms, irregular supplies, and non-standardized protocols.

**Discussion/Conclusion:**

The management of snakebites in West Africa is inconsistent and generally inadequate. The indirect costs to victims are largely overlooked. Improving access to appropriate antivenoms, training healthcare professionals, establishing structured surveillance systems, and engaging communities in awareness campaigns tailored to each local context are essential.

**Figure:**
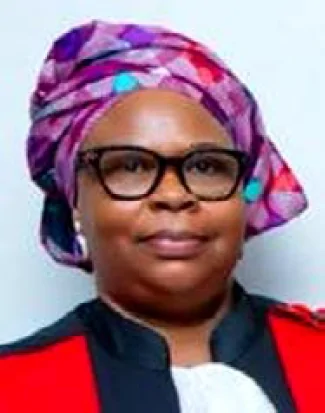

## Introduction

En Afrique intertropicale, les envenimations ophidiennes constituent une urgence médico-chirurgicale fréquente, quoique très fortement sousévaluée [9,12]. Les informations épidémio-logiques sur les morsures de serpents et les envenimations sont souvent incomplètes et généralement très localisées. Elles ne permettent que difficilement d’obtenir une estimation fiable de la situation dans un lieu donné [12]. Les populations rurales, tant en forêt qu’en savane, sont les plus exposées. Elles privilégient une prise en charge traditionnelle, ce qui entraîne des pratiques théra-peutiques inadaptées, voire à risque, et un retard de consultation fréquent [10,34]. À cela s’ajoute un accès aux soins difficile en raison de l’éloignement des centres de santé, de leur approvisionnement défectueux, notamment en antivenins, et d’une formation insuffisante du personnel de santé par ailleurs surchargé.

Le coût des antivenins est dissuasif, leur administration mal codifiée. Les ressources thérapeutiques disponibles ne favorisent pas la prise en charge adéquate bien que quelques études expérimentales et essais cliniques aient permis de confirmer l’efficacité des antivenins [16-18,35].

La Société africaine de venimologie (SAV) a été créée dans le but de coordonner les différentes initiatives pour une meilleure prise en charge des victimes d’envenimation en Afrique. Elle a élaboré un protocole de prise en charge des morsures de serpent devant faire l’objet de mises à jour régulières [14]. Dans les pays en voie de développement où le risque de morsure de serpent est majeur alors que les infrastructures sanitaires et les ressources sont limitées, ce travail vise à synthétiser les pratiques actuelles réalisées dans la prise en charge des morsures de serpent en Afrique de l’Ouest et les comparer aux recommandations standard de la SAV.

## Matériel et méthodes

Nous avons réalisé une revue narrative des stratégies de prise en charge des victimes de morsures de serpent dans certains pays d’Afrique de l’Ouest francophone, à savoir le Bénin, le Burkina Faso, la Côte d’Ivoire, la Guinée, le Mali, le Sénégal et le Togo. Dans ce contexte, l’analyse des études pertinentes s’avère particulièrement appropriée en raison de leur diversité et la complexité des facteurs en jeu. Nous avons adopté la démarche PICO, un outil méthodologique largement reconnu pour formuler clairement les questions de recherche et faciliter l’identification des études (Tableau I). Cette méthode permet de délimiter le sujet, de faire une synthèse critique et rigoureuse des données issues de multiples études en Afrique de l’Ouest, offrant une vue d’ensemble des différentes approches thérapeutiques. Elle permet aussi de définir les critères d’inclusion et d’orienter la discussion par une évaluation de l’impact des interventions mises en œuvre et ainsi d’identifier les facteurs associés à une meilleure prise en charge.

**Tableau I:** Découpage de la question de recherche en concepts et en mots clés selon le modèle PICO

P (Population)	I (Intervention)	C (Comparaison)	O (Outcomes)
Victimes de morsure de serpent au Bénin, Burkina Faso, Côte d'Ivoire, Guinée, Mali, Sénégal, Togo	Capacités de diagnostic, administration de l'antivenin selon le grade, surveillance clinique et biologique, prise en charge hospitalière: laboratoire, réanimation, chirurgie	Comparaison des approches de prise en charge des morsures de serpent en pratique et en théorie par rapport aux recommandations du protocole de prise en charge proposé par la SAV	Accès aux soins: délai de prise en charge, parcours thérapeutique, niveau de structure de santé, disponibilité antivenin / Diagnostic et prise en charge: gradation clinique, hospitalisation, administration antivenin, réanimation
MOTS-CLÉS UTILISÉS		MOTEURS DE RECHERCHE, ORGANISME	
Prise en charge - Morsure serpent - Afrique de l'Ouest		Google Scholar, Science Direct, OMS	
Snake bites - West Africa - Treatment - Management - Care - Evolution-trends		Pubmed	

La sélection des études identifiées a été réalisée en fonction de critères d’inclusion et d’exclusion prédéfinis. Les études incluses, quelle que soit la langue de publication, devaient a) porter sur des populations des pays choisis, b) évaluer les interventions de prise en charge des morsures de serpent et, c) fournir des données pertinentes pour l’analyse des méthodes thérapeutiques, l’accès aux soins, etc. Les études exclues étaient celles qui ne répondaient pas à ces critères ou dont la qualité méthodologique était jugée insuffisante. Les données pertinentes ont été incluses et enregistrées de manière standardisée suivant les articles, la revue des titres, puis le texte complet selon les critères d’inclusion et de non-inclusion. L’analyse a été faite suivant une synthèse narrative et qualitative des études retenues.

## Résultats

La recherche documentaire a permis d’identifier 1 568 publications potentiellement pertinentes. Après suppression des doublons et examen des titres et des résumés, 127 articles ont été retenus. Sur ces derniers, 80 répondaient aux critères d’inclusion prédéfinis et ont été soumis à une évaluation plus approfondie. Après lecture intégrale et minutieuse, huit articles ont finalement été choisis pour être inclus dans l’analyse. En complément de cette revue de littérature, une thèse a été jugée pertinente et a été intégrée à l’analyse. En définitive, neuf études (dont huit articles et une thèse) ont été retenues pour cette revue narrative (Fig. 1).

**Figure 1:**
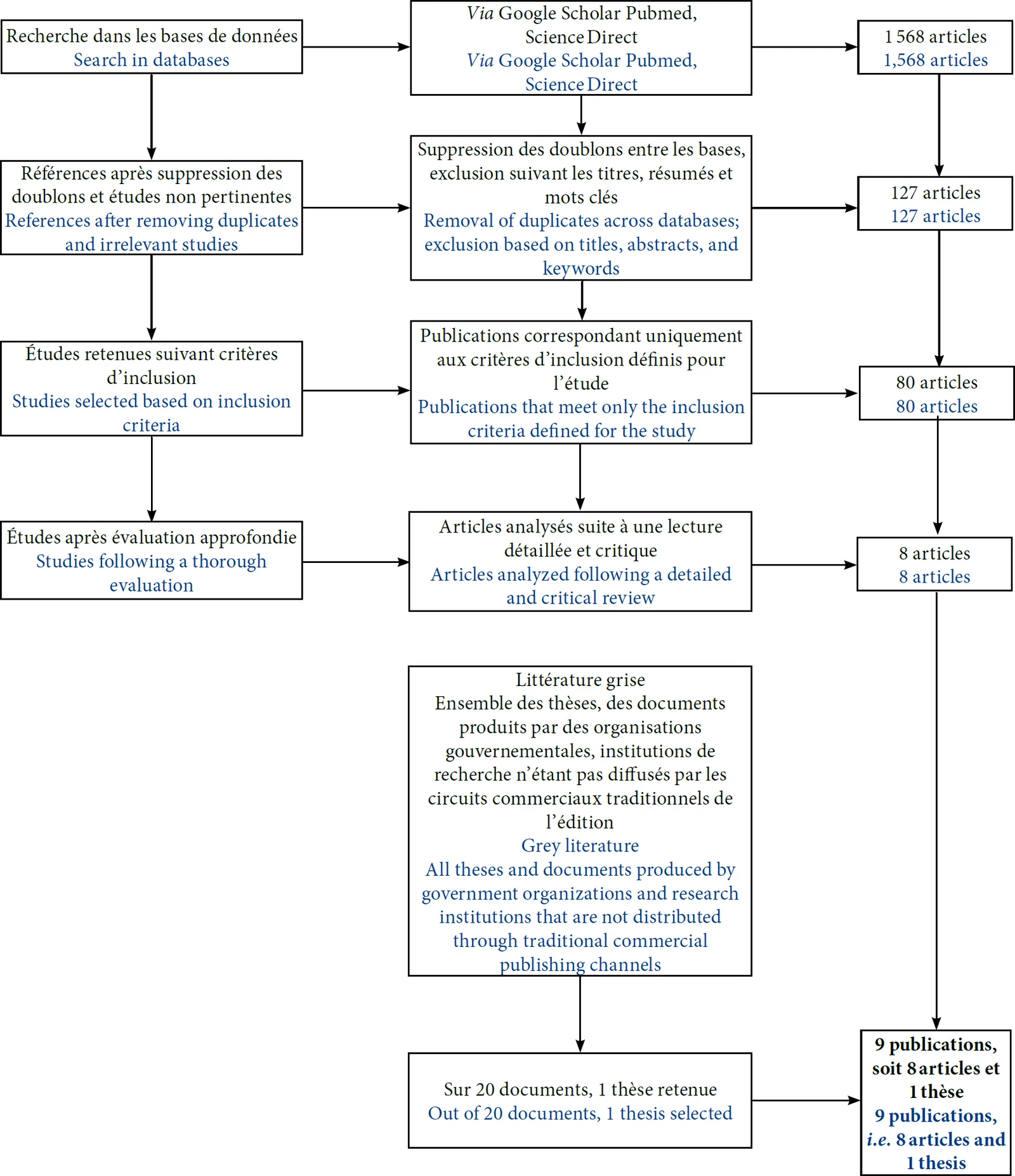
Étapes de sélection des études

Les études de Chippaux (2015) et de Sorge et Chippaux (2016) méritent d’être soulignées. La première a mis au point un algorithme décisionnel pour la prise en charge des morsures de serpent, fondé sur le protocole thérapeutique de l’OMS [14], tandis que la seconde a évalué la gravité des envenimations en classant différents syndromes [32]. Ces études rappellent quelques principes diagnostiques et thérapeutiques des morsures de serpent.

Ces deux études visaient à renforcer les connaissances de base en venimologie et à standardiser les protocoles de prise en charge des morsures de serpent validés par la SAV. Elles serviront ainsi de base comparative pour évaluer les différentes approches thérapeutiques et identifier les facteurs clés optimisant les stratégies de traitement des morsures ophidiennes. Les variables étudiées, comme domaines d’intervention concernant les patients et le système de santé, sont représentées sur un tableau comparatif (Tableau II).

**Tableau II:** Summary of the variables studied by country

	Bénin [35]	Burkina Faso [1]	Côte d'Ivoire [7]	Guinée [4]	Mali [22]	Sénégal [24,25,26]	Togo [6]
**Variables liées aux patients**							
Circonstances de morsure	81 % activités agricoles	43,28% activités agricoles - 29,05% domicile	93% abords des habitations broussailleuses	75% activités agricoles	activités agricoles	activités agricoles	75% activités agricoles
Sex-ratio (H/F)	1,6	1,45	1,1	1,8	1,7	1,5	1,43
Âge médian/moyen (ans)	27	23 ± 5,7	26	Adultes jeunes	29,7 ± 9,1	22	34 [23,5-42]
Patients ayant recours aux traitements ou soins traditionnels	72 %	51 %	61 %	45 %	47,36 %	80 – 90 %	NR
Retard de consultation/délai médian	7 heures [2-52]	3 heures [30 min- 7 jours]	4,2 ± 5,2 heures [40 min - 24 heures]	NR	8 ± 3,4 heures	6 heures [6-24]	> 24 heures
Évolution	3 % décès 2/57 cas	94,49% favorable 1,03% décès 5/560 cas	0 décès/75 / 0/75 deaths	18,2% décès 69/379 - 2,1% amputation	10,5% décès 2/19 cas séquelles	93,61 % favorable 4,54% décès 3/66 cas	59% favorable 9% décès 8/91 cas 18 complications
**Variables liées au système sanitaire**							
Structure d'accueil	Hôpital	Centre de santé	Réanimation	Structures sanitaires rurales	Centre de santé	Centre de santé/ hôpital	Hôpital
Disponibilité antivenin	Oui	Oui	Non	Non	Oui	Oui	Oui
Diagnostic/gradation	Oui	Oui	Oui	Oui	Oui	Oui	Oui
Prise en charge/Protocole SAV	100 % SAV	96,6% SAV	Application inconstante	Application inconstante	SAV	70,68% respect du protocole	SAV
Utilisation de l'antivenin	76%	Oui*	Indisponibilité	Indisponibilité	Oui*	Écart*	92%
TCTS/Biologie	Oui	Non	Non	Inconstante	Oui	Non	Oui
Traitement symptomatique/ Réanimation/Chirurgie	Symptomatique	Symptomatique	Symptomatique + chirurgie (2,6 %)	Symptomatique	Symptomatique	Symptomatique Chirurgie	Symptomatique
Surveillance clinique	Oui	NA	NR	Non	Oui	Inconstante	Oui
Durée d'hospitalisation	Médiane: 4 jours [2-5]	NR	Moyenne: 1,7 ± 1,1	NR	9,7 ± 5,1	Moyenne: 42 heures	NR

NR: Non renseigné NA: Non applicable * Pourcentage d'utilisation de l'antivenin non déterminé TCTS: temps de coagulation sur tube sec

L’analyse synthétique des données issues des études réalisées entre 1997 et 2023 dans plusieurs pays (Bénin, Burkina Faso, Côte d’Ivoire, Guinée, Mali, Sénégal, Togo) révèle un ancrage généralisé et persistant des pratiques traditionnelles. Dans toutes les localités étudiées, le recours initial aux tradipraticiens est prédominant. On note ainsi de manière constante des retards de consultation, de même que l’aggravation des lésions liées aux morsures, notamment par l’apparition d’infections, de complications nécrotiques ou de pertes fonctionnelles. Il ressort également de ces études le manque de surveillance clinique et biologique rigoureuse et la disponibilité irrégulière des antivenins.

## Discussion

Cette étude, bien que fondée sur une méthodologie rigoureuse, présente des limites, notamment l’hétérogénéité des sources (études hospitalières, thèses, rapports). Par ailleurs, l’absence de données récentes pour certains pays réduit la repré-sentativité temporelle.

Il s’agit aussi d’une étude restreinte aux pays francophones d’Afrique de l’Ouest où la problématique des envenimations est peu étudiée et où les communautés gardent souvent des considérations thérapeutiques ancestrales ou culturelles influençant le parcours des soins.

Au vu de ces aspects communs tels que les variables liées aux victimes de morsures de serpent, la 6e Conférence internationale sur les envenimations par morsures de serpent et piqûres de scorpion en Afrique s’est voulue être un tournant décisif dans la prise en charge des envenimations [16]. Elle a été organisée conjointement par la SAV et l’Institut Pasteur de Côte d’Ivoire en 2015 à Abidjan. Une diversité d’approches a été observée dans la gestion des morsures de serpents en Afrique de l’Ouest francophone. Ce qui justifie les initiatives nationales concrètes prises par les différents pays tels que le Burkina Faso, le Cameroun, la Côte d’Ivoire, le Sénégal et le Togo visant à améliorer l’accès aux soins et la disponibilité des antivenins. Cette hétérogénéité reflète les défis socio-économiques, logistiques et épidémiologiques propres à chaque pays, mais aussi une prise de conscience croissante de l’ampleur du problème de santé publique que représentent les envenimations ophidiennes.

### Accès aux soins

Le coût élevé du traitement hospitalier, en particulier lorsque l’antivenin est disponible mais non subventionné, représente un obstacle majeur à l’accès aux soins pour les populations rurales. Ce coût inclut, en plus de celui de l’antivenin, les frais d’hospitalisation, d’examens biologiques et de soins complémentaires. En l’absence de système de couverture sociale ou d’assurance maladie, ce fardeau économique peut pousser les familles vers les solutions traditionnelles perçues comme plus abordables [6,35].

Par ailleurs, l’indisponibilité fréquente des antivenins dans les centres de santé périphériques conforte cette tendance et renforce l’idée, au sein des communautés, que les centres de santé sont inefficaces face à ce type d’urgence. Les patients s’orientent ainsi vers les solutions traditionnelles, perçues comme plus accessibles ou mieux adaptées à leurs croyances et contraintes immédiates. Cette situation est particulièrement visible au Sénégal [24], où plusieurs antivenins ont été commercialisés, mais avec une disponibilité irrégulière et insuffisante, notamment dans les zones rurales. Le manque d’information claire et accessible au sein des communautés rurales sur la conduite à tenir en cas de morsure constitue un obstacle dans la mesure où la majorité des patients ignore les gestes proscrits. Ceci explique aussi le retard de consultation observé dans les différentes situations qui aggrave souvent le tableau clinique [6,20,22]. Lorsque l’antivenin est administré rapidement et dans des conditions adéquates, l’efficacité thérapeutique est très satisfaisante [24,25,28]. Cette immunothérapie est actuellement bien tolérée [8,19].

La disponibilité de l’antivenin au niveau des structures de soins opérationnelles contribue à une létalité relativement faible. Les décès, évitables dans la majorité des cas, sont presque toujours associés à un retard de prise en charge (> 12 h), à une non-administration d’antivenin, à des protocoles mal appliqués ou encore aux complications suites à des pratiques traditionnelles dangereuses. La proximité géographique des centres de traitement constitue un autre facteur décisif. Le Sénégal a instauré un maillage efficace avec des centres équipés jusque dans les zones rurales [26]. Dans la plupart des autres pays, l’antivenin n’est pas disponible dans les centres de santé, y compris dans de nombreux hôpitaux de référence, aggravant les délais de prise en charge. Cette situation peut conduire à des alternatives visant à favoriser l’accessibilité aux antivenins sans toutefois résoudre le problème du retard de consultation. Ainsi, l’Institut de Recherche en biologie appliquée de Guinée, à Kindia, clinique spécialisée dans la prise en charge des morsures de serpent, ou l’hôpital de Tanguiéta, au Bénin, attirent des patients provenant de très loin [2-5,35]. Malgré l’efficacité thérapeutique satisfaisante de l’antivenin en conditions optimales, la performance de la prise en charge est sévèrement entravée par l’hétérogénéité des protocoles thé-rapeutiques nationaux justifiée par des écarts et des lacunes substantielles dans la surveillance épidémiologique des envenimations. Cette surveillance reste en effet embryonnaire dans la majorité des pays étudiés : registres papier tenus dans quelques hôpitaux de référence et plateforme numérique inexistante. Consciente de cet obstacle majeur, l’OMS a inscrit l’envenimation ophidienne parmi les maladies tropicales négligées en 2017 et publié en 2019 une stratégie mondiale visant à réduire de 50 % la mortalité d'ici 2030. Le pre- mier pilier de cette stratégie consiste à renforcer la collecte et le partage de données. Un volet opérationnel est l'ouverture, en 2021, de la plateforme interactive « Snakebite Information and Data Platform » destinée à aider les États à informatiser leurs notifications.

Au Sénégal, le Centre antipoison (CAP) joue un rôle central dans la notification, la surveillance et la gestion des cas d'envenimation. Cependant, il reste confronté à des difficultés telles que la sous-notification des cas, l'accès restreint aux antivenins, le manque de données cliniques, et la méconnaissance des mécanismes de coordina- tion par le personnel de santé et les populations rurales. Le CAP a aussi contribué au financement d'études régionales telles que l'enquête d'incidence à Kédougou [26].

### Diagnostic et prise en charge

Bien que la majorité des pays d'Afrique de l'Ouest, membres de la SAV aient officiellement adopté le protocole standard de prise en charge des envenimations ophidiennes, son application effective sur le terrain demeure très inégale. Il en résulte une hétérogénéité des pratiques cliniques, avec des décisions thérapeutiques souvent laissées à l'appréciation individuelle, des doses d'antive- nin administrées insuffisantes et une évaluation thérapeutique rarement systématique. Nos résul- tats confirment cette variabilité, en mettant en évidence des écarts importants entre les pays étudiés en ce qui concerne l'évaluation clinique des patients, la disponibilité effective des antive- nins et la formation des professionnels de santé [17,24]. Ce manque de standardisation compromet l'efficacité globale de la prise en charge malgré la forte incidence des envenimations signalées [11]. Les résultats mettent encore en évidence trois obstacles majeurs : a) une dépendance marquée aux traitements traditionnels, qui retarde l'accès aux traitements spécifiques [15]; b) un manque de formation du personnel de santé, (phénomène confirmé par les études de Dabo et al. et Sidibé en 2023 [21,31]), qui explique la faible utilisation ou l'administration inadéquate des antivenins; c) le coût élevé prévisible du traitement hospi- talier, qui représente une barrière économique significative pour les populations rurales. Ce coût inclut antivenin, examens et hospitalisation, avec le cas échéant des pathologies ou des séquelles irréversibles [23,33,35,36] associées.

L’analyse comparative des approches nationales en Afrique de l’Ouest met en évidence plusieurs facteurs essentiels à une prise en charge efficace des envenimations ophidiennes nécessitant des recommandations formelles. De nombreuses recommandations ont été formulées lors de la 6è Conférence internationale sur les envenimations par morsures de serpent et piqûres de scorpions en Afrique, que les représentants de chaque pays se sont engagés à transmettre et à mettre en œuvre [16]. Ces engagements marquent un tournant stratégique pour une meilleure coordination de la lutte contre les envenimations ophidiennes en Afrique de l’Ouest.

Ces recommandations doivent être formulées dans l’espoir de faire émerger des réponses concertées, pragmatiques et adaptées. Ce travail se veut ainsi un socle de réflexion, mais aussi un levier d’action pour bâtir, finalement, une prise en charge des envenimations ophidiennes qui soit à la hauteur des enjeux humains qu’elles soulèvent.

Les autorités, le personnel de santé et la population sont ciblés conjointement:

- instaurer la déclaration obligatoire des cas;
- renforcer la formation du personnel de santé dans le cadre de la formation continue;
- renforcer la politique du médicament adaptée aux contextes nationaux, en privilégiant des antivenins de qualité et sûrs;
- mener des études épidémiologiques pour évaluer les besoins en antivenins;
- assurer l’accessibilité financière et géographique des antivenins par des financements diversifiés;
- appliquer le protocole thérapeutique standardisé, tel que préconisé par la SAV;
- sensibiliser la population pour une précocité de la consultation au niveau des structures sanitaires.

## Conclusion

Les morsures de serpent représentent un enjeu de santé publique majeur en Afrique subsaharienne dont l’ampleur reste largement sous-évaluée. Cette étude illustre une charge morbide, des complications et une létalité non négligeable, dont l’ampleur est largement favorisée par des retards de consultation, la difficulté d’accès aux soins et l’ancrage des pratiques traditionnelles souvent inadaptées. Elle permet d’apporter un éclairage actualisé et transversal sur cette problématique à travers l’analyse de plusieurs études régionales menées entre 1997 et 2023.

Des avancées notables et progressives ont pourtant été enregistrées ces dernières années, faisant suite à l’organisation de conférences structurantes, à l’image de celles du Sénégal en 2010, du Bénin en 2012, d’Abidjan en 2015 et celle en ligne coorganisée au Cameroun et au Bénin en 2021. Néanmoins, le déploiement de ces initiatives sur le terrain est freiné par des obstacles structurels persistants. Ces engagements doivent marquer un tournant stratégique pour une meilleure coordination régionale de la lutte contre les envenimations ophidiennes, étape majeure dans l’amélioration de la prise en charge des envenimations en Afrique. À travers ce travail se dessine un appel à renforcer l’équité dans l’accès aux soins et à replacer les réalités locales, contraintes logistiques, précarités sociales au cœur des stratégies sanitaires.

## Financement

Aucun.

## Contribution des auteurs et autrices

Lam Absa: initiation du manuscrit, revue de la littérature, rédaction, soumission, correction du manuscrit

Diallo Khardiata: revue de la littérature, correction du draft, relecture de l’article

Diallo Moussa: revue de la littérature Kane Arame: revue de la littérature Ndione Yacine : revue de la littérature

Diouf Coumba Ndofféne: relecture et correction du draft

Fall Mamadou: supervision, rédaction du draft et révision critique

## Déclaration de liens d’intérêt

Aucun lien d’intérêt n’a été déclaré.
