# Supplementary material for: A narrative review of strategies for managing snakebites in French-speaking West Africa
Source: Med Trop Sante Int. 2026 Aug 28;6(3):mtsi.v6i3.2026.834. [Article in French] doi: 10.48327/mtsi.v6i3.2026.834 (PMC13697905; doi:10.48327/mtsi.v6i3.2026.834)
Supplement: Related texts

# Research article: A narrative review of strategies for managing snakebites in French-speaking West Africa

## Introduction

In tropical Africa, snakebites are a common medical and surgical emergency, though they are significantly underreported [9,12]. Epidemiological data on snakebites and envenomation are often incomplete and highly localized. This makes it difficult to obtain a reliable assessment of the situation in a given area [12]. Rural populations in both forest and savanna regions are most at risk. These populations tend to favor traditional treatments, which can lead to inappropriate or risky therapeutic practices and frequent delays in seeking medical care [10, 34]. This problem is compounded by limited access to care due to distance from health centers, inadequate supplies—particularly of antivenom—and insufficient training of healthcare personnel, who are also overburdened.

The cost of antivenoms is prohibitive, and their administration is poorly standardized. Available therapeutic resources do not support adequate care, though a few experimental studies and clinical trials have confirmed the effectiveness of antivenoms [16-18,35].

The African Society of Venimology (ASV) was established to coordinate various initiatives aimed at improving care for snakebite victims in Africa. ASV has developed a snakebite management protocol that will be updated regularly [14]. In developing countries where the risk of snakebites is high but healthcare infrastructure and resources are limited, the goal of this study is to summarize and compare current snakebite management practices in West Africa with the ASV’s standard recommendations.

## Materials and methods

We conducted a narrative review of strategies for managing snakebite victims in several French-speaking West African countries: Benin, Burkina Faso, Côte d’Ivoire, Guinea, Mali, Senegal, and Togo. Given the diversity of the studies and the complexity of the factors involved, an analysis of relevant studies is particularly appropriate in this context. We adopted the PICO approach, a widely recognized methodological tool that helps to clearly formulate research questions and identify studies (Table I). This method enables us to define the scope of the topic, conduct a critical and rigorous synthesis of data from multiple West African studies, and provide an overview of the various therapeutic approaches. The PICO approach also enables us to define inclusion criteria and guide the discussion by assessing the impact of implemented interventions, thereby identifying factors associated with better care.

**Table I:** Breakdown of the research question into concepts and keywords according to the PICO model

P (Population)	I (Intervention)	C (Comparison)	O (Outcomes)
Snake bite victims in Benin, Burkina Faso, Côte d“Ivoire, Guinea, Mali, Senegal, and Togo	Diagnostic capabilities, administration of antivenom based on grade, clinical and laboratory monitoring, and hospital care: laboratory services, resuscitation, surgery	Comparison of the practical and theoretical approaches to snake bite management relative to the recommendations of the management ASV protocol	Access to Care: time to treatment, treatment pathway, level of healthcare facility, and availability of antivenom Diagnosis and management: clinical grading, hospitalization, antivenom administration, resuscitation
**USED KEYWORDS**		**SEARCH ENGINES, ORGANIZATION**	
Prise en charge - Morsure serpent - Afrique de l“Ouest		Google Scholar, Science Direct, WHO	
Snake bites - West Africa - Treatment - Management - Care - Evolution-trends		Pubmed	

The selection of the identified studies was based on predefined inclusion and exclusion criteria. Included studies, regardless of publication language, had to: a. Involve populations from the selected countries; b. Evaluate interventions for managing snakebites; c. Provide relevant data for analyzing therapeutic methods, access to care, etc. Studies were excluded if they did not meet these criteria or if their methodological quality was insufficient.

Relevant data were included and recorded in a standardized manner by first reviewing article titles, then reviewing the full texts according to the inclusion and exclusion criteria. The analysis was conducted as a qualitative and narrative synthesis of the selected studies.

## Results

The literature search yielded 1,568 potentially relevant publications. After removing duplicates and reviewing the titles and abstracts of the remaining publications, 127 articles were selected for further evaluation. Eighty of these met the predefined inclusion criteria and underwent a more in-depth evaluation. After thoroughly reading the full texts, eight articles were selected for the analysis. In addition to the literature review, one doctoral dissertation was deemed relevant and included in the analysis. Ultimately, nine studies (including eight articles and one dissertation) were selected for this narrative review (Fig. 1).

**Figure 1:** Study selection steps

The studies by Chippaux (2015) and Sorge and Chippaux (2016) are notable. The former established a decision-making algorithm for the management of snakebites based on the WHO [14] treatment protocol, and the latter assessed the severity of snake envenomations by grading various syndromes [32]. These studies highlight several diagnostic and therapeutic principles for snakebites.

These studies aimed to strengthen basic venimology knowledge and standardize snakebite management protocols validated by the ASV. Thus, they will serve as a basis for comparing different therapeutic approaches and identifying key factors that optimize treatment strategies for snakebites. The variables studied, such as areas of intervention related to patients and the healthcare system, are presented in Table II.

**Table II:** Summary of the variables studied by country

	Benin [35]	Burkina Faso [1]	Côte d'Ivoire [7]	Guinea [4]	Mali [22]	Senegal [24,25,26]	Togo [6]
**Patient-related variables**							
Circumstances of the bite	81% agricultural activities	43.28% agricultural activities - 29.05% home	93% near home - brush	75% agricultural activities	agricultural activities	agricultural activities	75% agricultural activities
Sex ratio (M/F)	1,6	1,45	1,1	1,8	1,7	1,5	1,43
Median/mean age (years)	27	23 ± 5,7	26	Young adults	29,7 ± 9,1	22	34 [23,5-42]
Patients seeking traditional treatments or care	72 %	51 %	61 %	45 %	47,36 %	80 – 90 %	NR
Delay in seeking care/median time to presentation	7 hours [2-52]	3 hours [30 min- 7 days]	4,2 ± 5,2 hours [40 min - 24 days]	NR	8 ± 3,4 hours	6 hours [6-24]	> 24 hours
Course of illness	3% mortality rate; 2/57 cases	94.49% favorable, 1.03% deaths, 5/506 cases	0/75 deaths	18.2% deaths 69/379 - 2.1% amputations	10.5% deaths, 2/19 cases with complications	93.61% favorable, 4.54% deaths, 3/66 cases	59% favorable, 9% deaths, 8/91 cases, 18 complications
**Variables related to the healthcare system**							
Treatment facility	Hospital	Health Center	Resuscitation	Rural Health Facilities	Health Center	Health Center/ Hospital	Hospital
Availability of antivenom	Yes	Yes	No	No	Yes	Yes	Yes
Diagnosis/triage	Yes	Yes	Yes	Yes	Yes	Yes	Yes
Management/ASV protocol	100% ASV	96.6% ASV	Inconsistent Application	Inconsistent Application	ASV	70.68% compliance with the protocol	ASV
Antivenom use	76%	Yes*	Unavailability	Unavailability	Yes*	Divergence*	92%
WBCT/laboratory tests	Yes	No	No	Inconsistent	Yes	No	Yes
Symptomatic treatment/resuscitation/surgery	Symptomatic	Symptomatic	Symptomatic Surgery (2.6%)	Symptomatic	Symptomatic	Symptomatic Surgery	Symptomatic
Clinical monitoring	Yes	NA	NR	No	Yes	Inconsistent	Yes
Length of hospital stay	Median: 4 days [2-5]	NR	Average: 1.7 ± 1.1	NR	9,7 ± 5,1	Average: 42 hours	NR

NR: Not reported NA: Not applicable

* Percentage of antivenom use not determined WBCT: Whole Blood Clotting Time

A comprehensive analysis of data from studies conducted between 1997 and 2023 in several West African countries (Benin, Burkina Faso, Côte d’Ivoire, Guinea, Mali, Senegal, and Togo) reveals a widespread and persistent reliance on traditional healing practices. In all the studied localities, initial recourse to traditional healers is predominant. Consequently, there are consistent delays in seeking medical care, which worsens bite-related injuries, particularly due to infections, necrotic complications, and functional impairment. The studies also highlight the lack of rigorous clinical and laboratory monitoring and the inconsistent availability of antivenoms.

## Discussion

Although this study is based on a rigorous methodology, it has limitations, notably the heterogeneity of the sources (hospital studies, theses, and reports). Furthermore, the lack of recent data for certain countries reduces its temporal representativeness.

Additionally, this study is limited to French-speaking countries in West Africa, where the issue of snakebite envenomation has received little attention, and where communities often adhere to traditional or cultural therapeutic practices that influence the course of care.

Considering these common factors, such as variables related to snakebite victims, the 6^th^ International Conference on Snakebite and Scorpion Sting Envenomation in Africa aimed to be a turning point in envenomation management [16]. The ASV and the Pasteur Institute of Côte d’Ivoire jointly organized it in Abidjan in 2015. This justifies the concrete national initiatives taken by various countries, such as Burkina Faso, Cameroon, Côte d’Ivoire, Senegal, and Togo, aimed at improving access to care and the availability of antivenoms. This heterogeneity reflects each country’s unique socioeconomic, logistical, and epidemiological challenges, as well as a growing awareness of the magnitude of the public health problem posed by snakebites.

### Access to care

The high cost of hospital treatment, particularly when antivenom is available but not subsidized, represents a major barrier for rural populations seeking care. In addition to the cost of antivenom, this cost includes expenses for hospitalization, laboratory tests, and complementary care. Without a social security system or health insurance, families may turn to traditional remedies, which are perceived as more affordable [6,35].

Furthermore, the frequent unavailability of antivenom in peripheral health centers reinforces this trend, strengthening the perception within communities that health centers are ineffective in dealing with this type of emergency. Patients thus turn to traditional remedies, which are perceived as more accessible or better suited to their beliefs and immediate constraints. This situation is particularly evident in Senegal [24], where several antivenoms are available but are irregularly and insufficiently supplied, especially in rural areas. A lack of clear, accessible information in rural communities about how to respond to a bite is an obstacle because the majority of patients are unaware of which actions to avoid. This also explains the delay in seeking medical care observed in various situations, which often worsens the clinical picture [6,20,22]. When antivenom is promptly and appropriately administered, it is highly effective [24,25,28]. This immunotherapy is currently well tolerated [8,19].

The availability of antivenom at operational healthcare facilities contributes to the relatively low case-fatality rate. The majority of deaths are preventable and are almost always associated with delayed treatment (>12 hours), failure to administer antivenom, poorly implemented protocols, or complications resulting from dangerous traditional practices.

Geographic proximity to treatment centers is another decisive factor. Senegal has established an effective network of well-equipped centers that extend into rural areas. In most other countries, however, antivenom is unavailable in health centers, including many referral hospitals, exacerbating delays in treatment. This situation may lead to alternative approaches aimed at improving access to antivenom, though it does not resolve the problem of delayed consultation. For instance, the Institut de Recherche en biologie appliquée de Guinée in Kindia—a clinic that specializes in treating snakebites—and the Tanguiéta Hospital in Benin attract patients from far away [2-5,35]. Although antivenom is therapeutically effective under optimal conditions, the effectiveness of care is severely hindered by the heterogeneity of national treatment protocols. This heterogeneity stems from substantial gaps and shortcomings in the epidemiological surveillance of snakebites. In fact, surveillance remains in its infancy in most countries: paper-based registries are maintained in a few referral hospitals, and there is no digital platform in place. In recognition of this significant challenge, the WHO classified snakebite as a neglected tropical disease in 2017 and published a global strategy in 2019 with the goal of reducing mortality by 50% by 2030. The first pillar of this strategy is strengthening data collection and sharing [15,18,30]. One operational component is the 2021 launch of the interactive “Snakebite Information and Data Platform,” which is designed to help countries digitize their reporting. In Senegal, the Poison Control Center (PCC) plays a central role in reporting, surveillance, and management of snakebite cases [27]. However, the PCC continues to face challenges, including underreporting of cases, limited access to antivenoms, a lack of clinical data, and a lack of awareness of coordination mechanisms among healthcare workers and rural populations. The PCC has also contributed to funding regional studies, such as the incidence survey in Kédougou [26].

### Diagnosis and management

Although the majority of West African countries that are members of the ASV have officially ad- opted the standard protocol for the management of snakebites, its actual implementation in the field remains highly inconsistent. This results in heterogeneity in clinical practices, with treat- ment decisions often left to individual judgment, insufficient doses of antivenom administered, and therapeutic evaluation rarely conducted sys- tematically. Our results confirm this variability, highlighting significant disparities among the countries studied regarding the clinical evaluation of patients, the actual availability of antiven- om, and the training of healthcare professionals [17,24]. This lack of standardization compromises the overall effectiveness of care despite the high incidence of reported snakebites [11].

The results further highlight three major obstacles: a) a marked reliance on traditional treatments, which delays access to specific treatments; b) a lack of training among healthcare personnel (a finding confirmed by studies by Dabo et al. and Sidibé in 2023 [21,31]), which explains the low use or inadequate administration of antivenoms; c) the predictably high cost of inpatient treatment, which represents a significant economic barrier for rural populations. This cost includes antivenom, diagnostic tests, and hospitalization, along with any associated conditions or irreversible sequelae [23,33,35,36].

A comparative analysis of national approaches in West Africa revealed several essential fac- tors for effectively managing snakebites, which require formal recommendations. During the 6th International Conference on Snakebites and Scorpion Stings in Africa, numerous recommen- dations were formulated, and representatives from each country committed to disseminating and implementing them. These commitments represent a significant step toward more coordi- nated efforts to combat snakebite envenomation in West Africa.

The recommendations aim to foster coordinated, pragmatic, and tailored responses. This work serves as a foundation for reflection and a cata- lyst for action to establish a system for managing snakebite envenomation commensurate with the human challenges it poses.

The recommendations target authorities, health-care workers, and the general public:

- implement mandatory case reporting;
- strengthen training for healthcare workers through continuing education;
- strengthen medication policies tailored to national contexts, prioritizing high-quality, safe antivenoms;
- conduct epidemiological studies to assess antivenom needs;
- ensure the financial and geographic accessibility of antivenoms through diversified funding;
- apply the standardized treatment protocol recommended by the ASV;
- raise public awareness to encourage early consultation at health facilities.

## Conclusion

Snakebites are a significant public health concern in sub-Saharan Africa, though the extent of the issue is often underestimated. This study high-lights the significant disease burden, complications, and mortality associated with snakebites, which are largely exacerbated by delays in seeking medical care, limited access to healthcare, and the prevalence of inappropriate traditional practices. The study provides an up-to-date, cross-sectional perspective on this issue by analyzing several regional studies conducted between 1997 and 2023.

However, significant progress has been made in recent years following landmark conferences in Senegal (2010), Benin (2012), Abidjan (2015), and an online conference co-organized by Cameroon and Benin (2021). However, implementing these initiatives is hindered by persistent structural obstacles. These commitments must mark a strategic turning point toward better regional coordination in the fight against snakebites—a major step toward improving snakebite case management in Africa.

This work emphasizes the need to strengthen equity in access to care and to consider local realities, logistical constraints, and social vulnerabilities when developing health strategies.

## Funding

None.

## Authors’ contributions:

Lam Absa: Manuscript initiation, literature review, manuscript drafting, submission, revision

Diallo Khardiata: Literature review, draft editing, and proofreading of the article

Diallo Moussa: Literature review Kane Arame: Literature review

Ndione Yacine: Literature review

Diouf Coumba Ndofféne: Proofreading and editing the draft

Fall Mamadou: Supervision, drafting, and critical review.

## Conflicts of interest

The authors declare no conflicts of interest.
